# *Leishmania major* Self-Limited Infection Increases Blood Cholesterol and Promotes Atherosclerosis Development

**DOI:** 10.1155/2013/754580

**Published:** 2013-04-28

**Authors:** Luciana R. Fernandes, Ana Cecília C. Ribeiro, Marcela Segatto, Luís Felipe F. F. Santos, Joana Amaral, Luciane R. Portugal, Jacqueline I. A. Leite

**Affiliations:** ^1^Departamento de Bioquímica e Imunologia, Universidade Federal Minas Gerais, 31270-901 Belo Horizonte, MG, Brazil; ^2^Centro Universitário, Av. Prof. Mário Werneck, 1685-Estoril, 30455-610 Belo Horizonte, MG, Brazil

## Abstract

*Leishmania major* infection of resistant mice causes a self-limited lesion characterized by macrophage activation and a Th1 proinflammatory response. Atherosclerosis is an inflammatory disease involving hypercholesterolemia and macrophage activation. In this study, we evaluated the influence of *L. major* infection on the development of atherosclerosis using atherosclerosis-susceptible apolipoprotein E-deficient (apoE KO) mice. After 6 weeks of infection, apoE KO mice exhibited reduced footpad swelling and parasitemia similar to C57BL/6 controls, confirming that both strains are resistant to infection with *L. major*. *L. major*-infected mice had increased plasma cholesterol levels and reduced triacylglycerols. With regard to atherosclerosis, noninfected mice developed only fatty streak lesions, while the infected mice presented with advanced lesions containing a necrotic core and an abundant inflammatory infiltrate. CD36 expression was increased in the aortic valve of the infected mice, indicating increased macrophage activation. In conclusion, *L. major* infection, although localized and self-limited in resistant apoE KO mice, has a detrimental effect on the blood lipid profile, increases the inflammatory cell migration to atherosclerotic lesions, and promotes atherogenesis. These effects are consequences of the stimulation of the immune system by *L. major*, which promotes the inflammatory components of atherosclerosis, which are primarily the parasite-activated macrophages.

## 1. Introduction

*Leishmania major* is a protozoan parasite transmitted by sandflies of the genus *Lutzomyia* that inject the promastigote form into the dermis of the host. Once injected, the parasite is rapidly enclosed by phagocytic cells and transforms into the replicative intracellular amastigote form [1]. In immunocompetent hosts, such as C57BL/6 mice, *L. major* infection is a self-contained cutaneous lesion that elicits a Th1 immune response. In infected mice, the immune cells (macrophages, dendritic cells, natural killer cells, and T cells) produce cytokines and bioactive molecules, such as IFN-**γ**, IL-12, and nitric oxide (NO), which act against the protozoan [1]. 

Atherosclerosis is a chronic inflammatory disease associated with a high level of total cholesterol and proatherogenic lipoproteins (VLDL, IDL, and LDL), a prothrombotic status, and a Th1-polarized immune response [2, 3]. Macrophage and endothelial cell activation by atypical lipoproteins and proinflammatory cytokines induces the production of adhesion molecules, cytokines and chemokines, and causes oxidative stress [4]. Among atherosclerosis risk factors, infection plays an important role in the perpetuation of chronic inflammation at the atherosclerotic site [5, 6]. Several studies suggest that pathogens, such as bacteria, viruses, and protozoa, contribute to the atherogenesis process [7–11]. The increased inflammation caused by these pathogens promotes macrophage activation and migration to the atheroprone site. Alternatively, proatherogenic status may be attributed to the systemic oxidative stress induced by infection, which enhances lipoprotein or endothelium oxidation. Pathogens involved in atherosclerosis development usually induce a systemic infection instead of a localized infection such as an *L. major* infection. However, even localized infections, such as odontologic ones, may be associated with the development of atherosclerosis [12].

Apolipoprotein (apo) E is a component of chylomicron and very low-density lipoproteins (VLDL) and mediates the uptake of these lipoproteins and possesses anti-inflammatory and antioxidant effects [13]. ApoE KO mice have impaired clearance of VLDL and chylomicrons from the blood, which results in hypercholesterolemia and favors the development of atherosclerotic lesions [11]. ApoE also has anti-inflammatory and antioxidative properties, and its absence is partially responsible for the higher inflammatory profile of apoE-deficient animals compared to the wild-type control, C57BL/6 mice [14]. ApoE KO mice spontaneously develop atherosclerosis even when fed a normolipidemic diet [15]. The atherosclerotic lesions of apoE KO mice exhibit a similar distribution, microscopic appearance, and cellular composition to those found in humans. The atherosclerotic lesions are characterized by endothelial dysfunction, macrophage, and T cell infiltration and production of proinflammatory cytokines, such as TNF-*α*, INF-*γ*, and IL-6, which indicates a typical Th1 polarized immune response [2].

This work aimed to determine if a self-limited *L. major* infection would be sufficient to modify the lipid metabolism and promote the development of atherosclerosis similar to what occurs with systemic infections.

## 2. Material and Methods

This protocol was approved by the Animal Care Committee of Universidade Federal de Minas Gerais (CETEA #147/05).

### 2.1. Mice and Diet

Twenty-eight 11-week-old female apoE KO mice were separated into control (noninfected) and *Leishmania major *(*L. major*) infected groups. For the background control, twenty wild-type C57BL/6 mice were divided into control (noninfected) or *L. major *infected groups. Animals of both strains were equally distributed according to weight and blood cholesterol and were fed on AIN-93G standard diet [15].

### 2.2. *Leishmania major* Infection

The mice were infected with *L. major* (clone WHO MHOM/IL/80/Friedlin). The inoculum was prepared under sterile conditions from five-day *L. major* cultures, which corresponds to the stationary growth phase rich in the promastigote metacyclic infectious forms. The medium containing *L. major* was centrifuged, and the pellet was resuspended in 1 mL of PBS. Approximately 1 × 10^6^ parasites were inoculated into the left footpad, and the right footpad was used as a control after inoculation with PBS as previously described [16]. The footpad was measured weekly with a caliper, and the amount of swelling was calculated as the difference between the measures of the infected (left) and control (right) footpads.

The animals' body weight and food intake were measured weekly for 6 weeks after infection. All groups received the same amount of food to avoid confounding the experiment with different amounts of nutrients. After 6 weeks and an overnight fast, the mice were euthanized under anesthesia. Plasma, footpads, and tissues were collected for subsequent analyses. The infected footpad was weighed without the skin and used for parasite quantification as previously described [16].

### 2.3. Lipids Assay

The total cholesterol, cholesterol in high density lipoprotein (HDLc) form, and triacylglycerols in the plasma were measured using commercial kits (Doles, Brazil) as previously described [17]. The amount of non-HDLc (that represents the atherogenic fractions VLDLc and IDLc) in the plasma was determined by calculating the difference between the total cholesterol and the HDLc. 

The liver was washed in PBS and dried on filter paper. The contents of the cecum were separated from the lumen. The hepatic and cecal lipid extractions were performed as previously described by Folch et al. [18].

### 2.4. Histological Analysis

For the analysis of atherosclerosis, the aortic valves were washed with PBS, gently perfused with 10% neutral buffered formalin, and embedded in paraffin. The analysis was performed on 10 samples per group. The specimens were fixed in 3.7% paraformaldehyde and processed in paraffin. Briefly, every consecutive section (10 *μ*m thick) through the aortic valve (300 *μ*m) was taken and stained with hematoxylin and eosin [19]. The sections used for immunofluorescence were deparaffinized by 2 xylene washes (30 min) and then transferred to 3 washes (15 minutes each) of ethanol at concentrations of 100%, 85%, and 70%. The atherosclerotic lesion area was quantified using the Image Pro Plus software (Media Cybernetics, USA) from the sum of the atherosclerotic lesion areas obtained from the selected 10 sections, spaced 20 *μ*m apart, as previously described [11]. The inflammatory cells per field were counted automatically by the Image Pro Plus software (*n* = 5 mice, 3 nonconsecutive sections per animal) [11].

### 2.5. *L. major *Parasitism

The DNA in liver and spleen was extracted with proteinase K (Qiagen USA) according to the manufacturer's instructions. A PCR analysis was conducted [20]. Briefly, 2 *μ*L of DNA sample was added to 1.2 *μ*L of Taq polymerase buffer, 1.0 *μ*L of dNTPs (2.5 mM), 1.0 *μ*L of primers, 0.05 *μ*L of Taq polymerase, and 5.75 *μ*L of Milli-Q sterile H_2_O. The samples were placed in a thermocycler and the amplification conditions were as follows (35 cycles): denaturation at 94°C for 1 min, annealing at 54°C for 1 min, elongation at 72°C for 1.5 min, and extension at 72°C for 10 min. The PCR products were then subjected to gel electrophoresis polyacrylamide and visualized by silver staining. The sequences of the primers used, which correspond to the minicircle kDNA [20], are Uni 21-5′GGGGTTGGTGTAAAATAAGGCC 3′ and LmJ4-5′CTA.

### 2.6. Immunofluorescence Staining

Cross-sections of the aortic roots were blocked with 4% (wt/vol) BSA in PBS. Next, sections were incubated for 1 h at 22°C with FITC-goat antibody to mouse CD11b (0.2 mg/mL, 1 : 200 dilution; Santa Cruz Biotechnology, USA). Sections stained with FITC IgG anti-goat antibody served as negative controls. Sections were mounted with Vectashield medium and were visualized with an Axioscope 2 Plus fluorescence microscope (Carl Zeiss).

### 2.7. RT-PCR

The popliteal lymph node was homogenized using the TH-01 (OMNI-INC). The total RNA from the lymph node and aortic valve was extracted using the TRIzol reagent according to the manufacturer's protocol. The reverse transcription was performed using 2 *μ*g of the total RNA, 200 U of the reverse transcriptase, 2.5 *μ*L of the 5X RT buffer, 1.8 *μ*L of the 10 mM dNTPs, 0.2 *μ*L of the 10000 U/mL RNasin, and 1.0 *μ*L of the 50 *μ*M oligo dT. The temperature settings for this reaction were 70°C for 5 min, on ice for 2 min, 42°C for 60 min, 70°C for 15 min, and 4°C for the final step. The resulting cDNA was used for real-time PCR as described later. The specific primers were designed using the Primer Express software and synthesized by IDT. Real-time PCR was carried out on a StepOne sequence detection system (Applied Biosystems) using the Power SYBR Green PCR Master Mix (Applied Biosystems). The dissociation curve indicated that only one product was obtained in each reaction. The relative levels of gene expression were determined using the ΔΔ cycle threshold method as described by the manufacturer, in which data for each sample is normalized to the *β*-actin expression and the data are shown as the relative expression (the fold increase over the control). The PCR results were analyzed with the SDS 2.1 software (Applied Biosystems), and the amount of mRNA of each gene of interest was normalized to the amount of the murine *β*-actin gene. mRNA expression levels were calculated as the fold difference relative to the housekeeping gene by the formula: relative expression = 2^−(CT  [target  gene]−CT  [*β*-actin-1])^.

 The sequences of the primers used are as follows:

 VCAM-1: 5′-CCCCAAGGATCCAGAGATTCA-3′ and 5′-ACTTGACCGTGACCGGCTT-3′;
 CD36: 5′GTACAGATTTGTTCTTCCAGCCAAT-3′ and 5′-TCAGTGCAGAAACAATGGTTGTC-3′;
 MCP1/CCL2: 5′-AGGAAGATCTCAGTGCAGAG-3′ and 5′-AGTCTTCGGAGTTTGCCTTTG-3′;
 
*β*-actin: 5′-CTGCCTGACGGCCAAGTC-3′ and 5′-CAAGAAGGAA GGCTGGAAAAGA-3′;
 IL-6: 5′-ACAACCACGGCCTTCCCTACTT3′ and 5′-CACGATTTCCCAGAGAACATGTG-3′;
 IFN-*γ*: 5′-TGGCTCTGCAGGATTTTCATG-3′ and 5′-TCAAGTGGCATAGATGTGGAAGAA-3′;
 IL-10: 5′-GGTTGCCAAGCCTTATCGGA-3′ and 5′-ACCTGCTCCACTGCCTTGCT-3′;
 IL-4: 5′-ACAGGAGAAGGGACGCCAT-3′ and 5′-GAAGCCCTACAGACGAGCTCA-3′;
 LmjF36.5520: 5′-GGCCATCACCACAAACAGAG-3′ and 5-GCTCAGGTCATACAAGGGGA-3′.

### 2.8. Statistical Analyses

The data were initially analyzed by the Kolmogorov-Smirnov test to check their symmetry and by the Mann-Whitney or Student's *t*-test using GraphPad Prism, version 5.00, for Windows, GraphPad Software, San Diego, CA, USA. A *P* value of <0.05 was considered significant.

## 3. Results

### 3.1. *L. major *Infection in ApoE KO and Wild-Type Control C57BL/6 Mice

**Figure 1:**
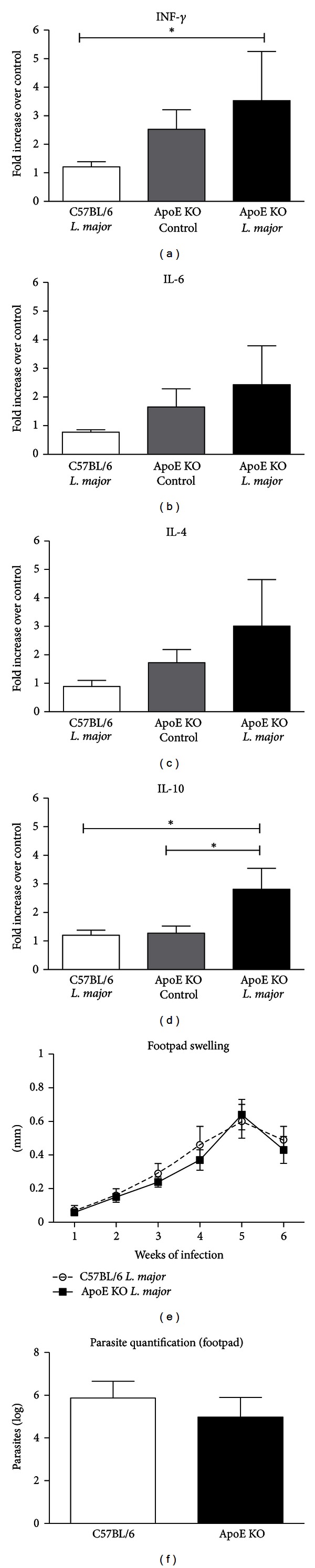
The infection profile of *L. major *in apoE KO and C57BL/6 animals infected with 1 × 10^6^ parasites (clone WHO MHOM/IL/80/Friedlin) into left footpad. (a to d) Cytokine (IL-4, IL-10, TNF-*α*, and IL-6) mRNA transcripts in the popliteal lymph nodes of apoE KO control, apoE KO *L. major*-infected, and C57BL/6 *L. major*-infected (*n* = 4/group) mice at the 6th experimental week. Values of average and standard error of cytokine mRNA transcripts in C57BL infected, apoE KO control, and apoE KO infected were, respectively, IL-4 = 0.89 ± 0.20; 1.72 ± 0.46 and 3.01 ± 1.6; IL-10 = 1.2 ± 0.12; 1.28 ± 0.24 and 2.81 ± 0.7; IFN*γ* = 1.20 + 0.82; 2.53 ± 0.63 and 3.52 + 1.7; IL-6 = 0.78 ± 0.80; 1.65 ± 0.63 and 2.4 ± 1.3. (e) The infection curve of apoE KO and C57BL/6 *L. major* infected mice. The results are expressed as the average of the footpad swelling calculated by the difference between the measures of the infected (left) and control (right) footpads. Number of mice: *n* = 11 in apoE KO and *n* = 7 in C57BL/6 groups for each week. (f) Footpad parasite quantification from apoE KO and C57BL/6 mice infected with *L. major* 6 weeks after infection. The results are expressed as the average of the log of the total parasites from apoE KO (*n* = 11) and C57BL/6 (*n* = 7) mice. Bars = average and vertical lines = standard error. *P < 0.05.

Our first aim was to confirm that *L. major* causes a self-limited infection in apoE KO mice similar to that observed in the wild-type control C57BL/6 mice. The results showed that after 6 weeks of infection, although apoE KO mice presented higher levels of the cytokines TNF alpha and IL-10 (Figures 1(a) to 1(d)), these mice controlled parasite load and footpad swelling similarly to C57BL/6 mice (Figures 1(e) and 1(f)). Therefore, only apoE KO animals were used for further experiments. 

There were no differences in the food intake between apoE experimental and control groups. As expected, *L. major* infection did not cause a significant weight loss in these animals (Table 1).

### 3.2. Lipid Profile

**Figure 2:**
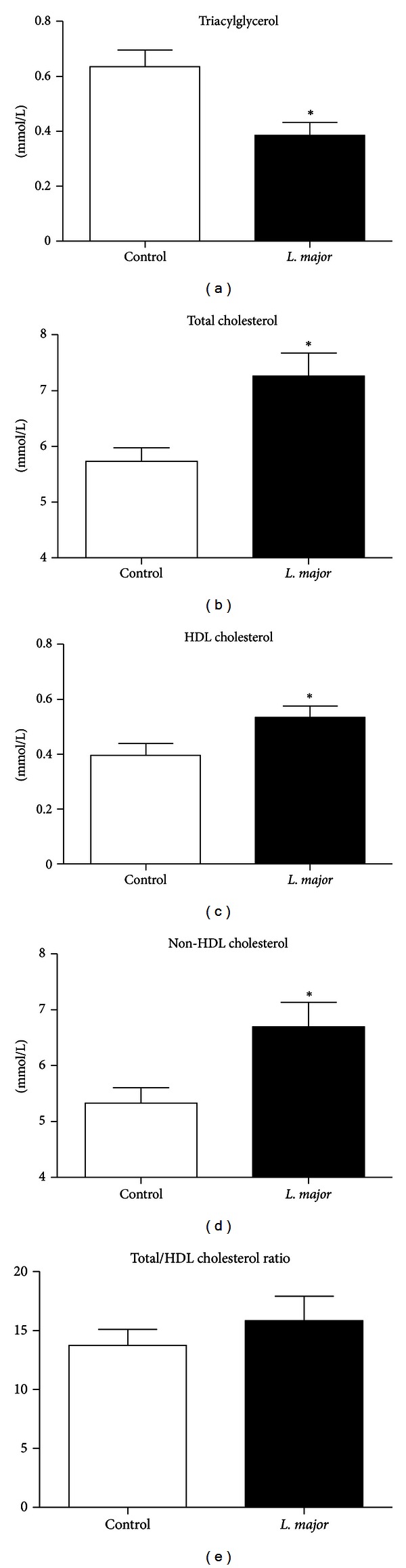
Blood lipid profile of noninfected (*n* = 9) and *L. major*-infected (*n* = 12) apoE KO mice 6 weeks after the inoculation of 1 × 10^6^ parasites (clone WHO MHOM/IL/80/Friedlin) into left footpad. (a) Total triacylglycerols; (b) total cholesterol; (c) high density lipoprotein (HDL) cholesterol; and (d) non-HDL cholesterol (sum of cholesterol in low density (LDL), intermediate density (IDL) lipoproteins, and remnant chylomicron), calculated as the difference between cholesterol and HDL cholesterol. Bars = average and vertical lines = standard error. *P < 0.05.

*L. major* infection was associated with increased serum cholesterol due to an increase in both HDL and non-HDL fractions (Figure 2). These alterations in the lipid profile were not due to differences in the hepatic cholesterol content or cecal excretion because the levels of these were similar between groups (Table 1).

*L. major* infection resulted in a reduction in the blood and hepatic triacylglycerol content compared to the controls (Figure 2(a) and Table 1), highlighting the possible influence of *L. major* on triacylglycerol metabolism. With regard to blood cholesterol, *L. major* infection was associated with increased serum total cholesterol due to an increase in both HDL and non-HDL fractions (Figures 2(b) to 2(d)), highlighting the influence of this parasite also on plasma cholesterol levels. 

### 3.3. Atherosclerotic Lesions

**Figure 3:**
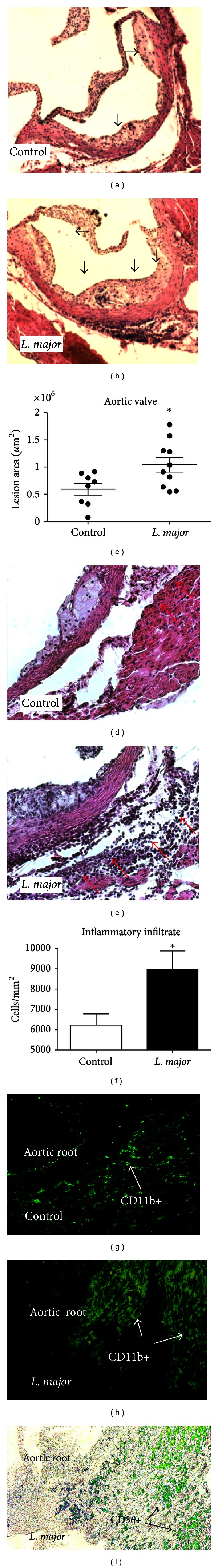
Atherosclerotic lesions and inflammatory infiltrate in apoE KO mice infected or noninfected (control) with *L. major* for 6 weeks. (a, b) Histology of the aorta from noninfected (a) and *L. major-*infected (b) apoE KO mice, 6 weeks after infection. (c) Atherosclerosis lesion area in aortic valve of noninfected (Control) and *L. major*-infected apoE KO mice. The results represent the average of the lesion area (*μ*m^2^) in the aortic valve of animal from the control (*n* = 7) and the infected groups (*n* = 10), 6 weeks after infection. The major lines represent the means and the minor lines represent the standard error. *P = 0.05. (d, e) Inflammatory infiltrate around the atherosclerotic lesion in aortic valve of non-infected (d) and *L. major-*infected (e) apoE KO mice. (f) Average number of cells/mm^2^ of aortic valve of non-infected (*n* = 5) and infected (*n* = 5) apoE KO mice 6 weeks after infection. The bars represent the average and vertical lines represent standard error, *P < 0.05. (g, h) Immunofluorescence: CD11b FITC conjugated positive cells in aortic root of control (g) and *L. major-*infected (h) mice. (i) Anti-CD36 antibody Alexa488 conjugated in aortic valve of *L. major*-infected group. In (a), (b), (d), and (e), the sections were stained with H&E. Image magnification: 20x in (a), (b), (g), and (h) and 100x in (d), (e), and (i).

Animals infected with *L. major* presented larger and more developed atherosclerotic lesions when compared to the controls (Figures 3(a), 3(c), and 3(e)). In the control group, atherosclerotic lesions were formed by fatty streaks and several layers of foam cells. On the other hand, in the infected animals, an intense inflammatory infiltrate and necrotic areas with deposition of cholesterol crystals (characteristics of an advanced atherosclerotic lesion) were frequently found (Figures 3(b), 3(d), and 3(f)). The inflammatory infiltrate was composed by cells of the innate immune system, including monocytes and macrophages as suggested by the higher number of CD11b positive cells around the atherosclerotic lesion of infected mice (Figures 3(g) and 3(h)).

### 3.4. Expression of VCAM-1, MCP1/CCL2, and CD36 in the Aortic Valve

The expression of the adhesion molecule VCAM-1, the monocyte chemotactic protein MCP-1/CCL2, and the scavenger receptor CD36 was also assessed in atherosclerotic lesions. 

**Figure 4:**
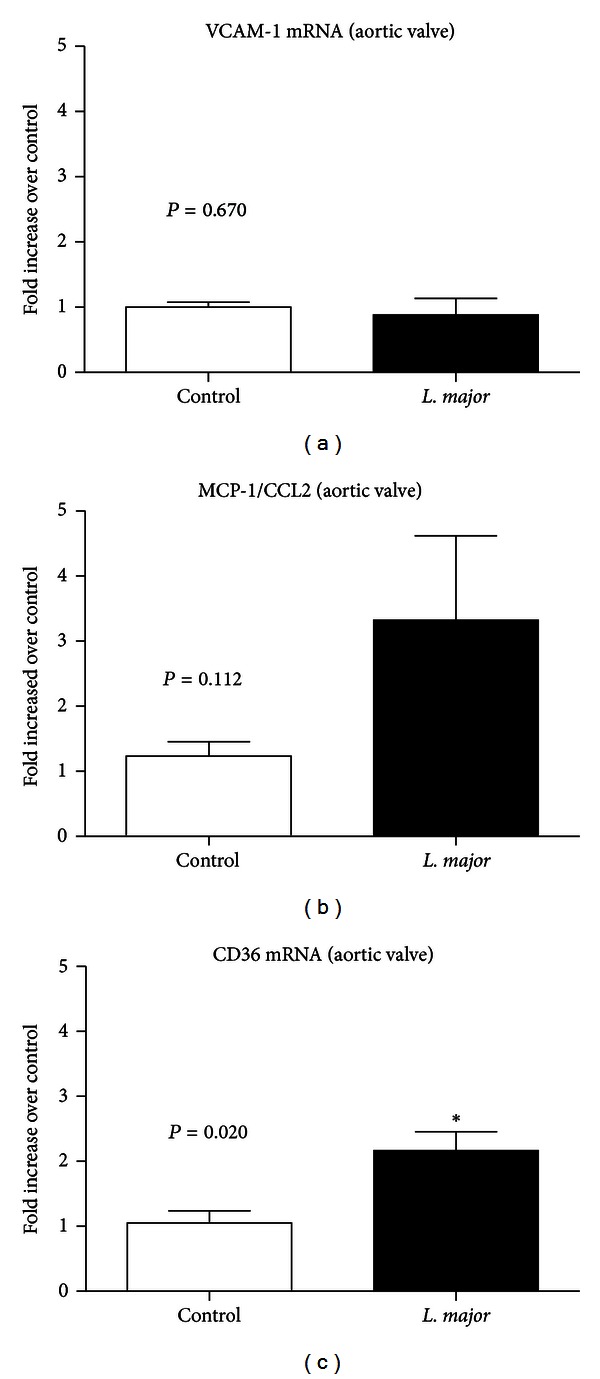
Relative increase of (a) VCAM-1, (b) MCP1/CCL2, and (c) CD36RNA expression in the aortic valve of non-infected and infected apoE KO mice, 6 weeks after infection. Bars = average and vertical lines = standard error, *n* = 4/group. *P < 0.05. The values of apoE KO control and *L. major* groups were, respectively, VCAM = 1.01 ± 0.07 and 0.89 ± 0.21; CD36 = 1.05 ± 0.19 and 2.16 ± 0.20; MCP-1 = 1.22 ± 0.22 and 2.75 ± 0.83.

Confirming the histological data, the results showed that CD36 expression was significantly increased in the *L. major* group (*P* = 0.02). There was a tendency for increased (*P* = 0.11) MCP1/CCL2 expression, and there were no changes in VCAM 1 (*P* = 0.67) expression (Figure 4).

### 3.5. *L. major* DNA in Peripheral Tissues

**Figure 5:**
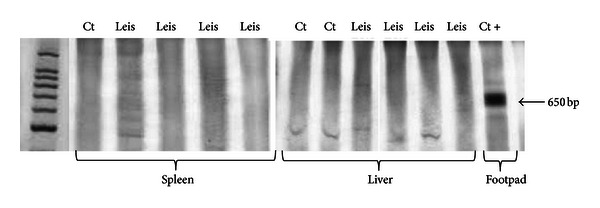
PCR amplification of *L. major* DNA in the spleen and liver of non-infected and *L. major-*infected apoE KO mice. First line: molecular weight marker; last line: positive control DNA from an *L. major*-infected footpad. Ct = apoE KO control mice; Leis- apoE KO *L. major*-infected mice.

Spleen and liver were tested for the presence of *L. major* DNA. The parasite was not detected in the spleen or liver of mice from both groups, suggesting the absence of viable parasites in these organs (Figure 5).

## 4. Discussion

To our knowledge, this is the first study investigating the effect of *Leishmania* infection on atherogenesis. A previous study [21] using apoE KO mice analyzed the influence of genetic and high-fat/cholesterol diet-induced dyslipidemia on T cell and dendritic cell (DC) responses to *L. major* infection* in vivo *and, mainly, *in vitro*. Although using the same mouse model used in the present study, the authors did not analyze atherosclerosis development and lipid profile. Moreover, cytokine determinations were done mainly *in vitro*, after parasite antigen restimulation. Finally, the resolution of *L. major* infection in C57BL/6 mice (measured by footpad swelling) started only after 10 weeks of infection while in ours and other studies [22–24] it occurred after 5 weeks of infection.

Our results show that *L. major* infection modifies blood lipid metabolism, resulting in the increase of total cholesterol and its fractions. In addition to the hypercholesterolemia, *L. major* infection also interfered with the metabolism of triacylglycerols, reducing their levels in the blood and liver. Despite the reduction in blood triacylglycerols, we found larger and more advanced atherosclerotic lesions in the aortic valves of infected mice. 

Our previous studies showed that *T. gondii*, which induces a strong systemic inflammation, promotes atherogenesis in apoE KO mice [11]. Because *L. major* infection is self-limited in mice, we expected that *L. major* would have little influence on atherosclerosis development compared to *T. gondii* infection. However, our results suggest an important role for *L. major* infection in macrophage activation and atherosclerosis development. 

Several mouse strains, including C57BL/6, effectively control *L. major* infection, while others, such as the BALB/c strain, develop progressive damage to the site of infection and systemic disease [25]. Our results indicate that apoE KO animals present the same pattern of resistance to *L. major *infection as that observed in the C57BL/6 animals, as determined by similar degrees of parasite load and footpad swelling. However, the pattern of cytokine expression after 6 weeks of infection shows a strong tendency to be higher in ApoE KO infected mice compared to the ApoE control and the C57BL/6 infected groups. This higher inflammatory status of ApoE KO mice has been previously presented as a consequence of the absence of anti-inflammatory effects of apoE as well as the proinflammatory stimulus of hypercholesterolemia and oxidized lipoproteins [26]. 

*L. major* infection leads to hypercholesterolemia associated with a reduction in blood and hepatic triacylglycerols without changes in cholesterol excretion. This pattern of lipid alteration was not found during a *T. gondii* infection [11], which reduces cholesterolemia with no alterations in the level of triacylglycerols in the blood. These different findings reflect the specific metabolic characteristics of each protozoan with regard to their use of host lipids. *T. gondii* is unable to produce cholesterol on its own. Thus, it is dependent on host cholesterol taken from the blood. Additionally, *T. gondii* causes a more severe infection that induces a reduction in food intake and weight loss in the mice. One of the consequences of this is the preferential use of acetyl CoA to supply energy, reducing its availability to the cholesterol synthesis pathway [27]. Both factors contribute (host cholesterol uptake and acetyl-coA redirection) to the reduction of cholesterolemia during a *T. gondii infection*. *Leishmania*, on the other hand, is able to synthesize its own cholesterol [28] but is dependent on host fatty acids, an important source of energy for amastigotes [29]. It is possible that *L. major* acquires fatty acids from triacylglycerol-rich lipoproteins, particularly IDL, which is the primary lipoprotein in apoE KO mice, resulting in the decrease of circulating triacylglycerol from lipoproteins in infected mice. On the other hand, the reduction of triacylglycerol could be related to the weight loss and a catabolic state. Independently of the cause, the reduction of the blood triacylglycerols stimulates the rapid conversion of the IDL into the more atherogenic, small, and dense LDL [30], which could explain the higher cholesterol and atherogenic fractions in the *L. major* group [31, 32]. 

*L. major* infection also stimulated the migration of inflammatory cells to the atherosclerotic lesion site. Aside from the hypercholesterolemia, these results could be explained by the oxidative stress and endothelial dysfunction resulting from inflammation [33], which leads to rapid migration of immune cells to the atherosclerotic lesion, where they accelerate atherogenesis. Moreover, *L. major *infected leukocytes could egress from the footpad tissue or draining lymph node and, following chemotactic stimulus, and migrate to other inflammatory regions such as the atherosclerotic site. Macrophages are connected to the progression of atherosclerosis through the production of inflammatory cytokines, which activate endothelial mediators and prothrombotic factors that are important in the atherosclerosis development. The increased movement of *L. major*-activated leukocytes to the site of atherosclerosis is suggested by the higher inflammatory infiltration and the higher expression of CD36, the major macrophage scavenger receptor for abnormal (oxidized) LDL. The introduction of minimally oxidized LDL into the intimae of arteries causes endothelial activation and release of leukocyte chemotactic factors that attract macrophages and other immune cells, which aggravates the inflammation and enhances plaque formation. In our study, parasite DNA was not found in the spleen or liver, refuting the hypothesis of visceralization. 

In conclusion, *L. major* infection, although localized and self-limited in resistant apoE KO mice, has a detrimental effect on the blood lipid profile, increasing the inflammatory cell migration to atherosclerotic lesions and accelerating atherogenesis. The latter is the consequence of the increase of the inflammatory component of atherosclerosis that could be trigged by the parasite-activated macrophages. 

## Figures and Tables

**Table 1:** Final body weight, food intake, and cecal and hepatic lipids of non-infected (control) and *L. major*-infected (*L. major*) apoE KO mice 6 weeks after the inoculation of 1 × 10^6^ parasites (clone WHO MHOM/IL/80/Friedlin) into footpad.

Parameter	Control ApoE KO	*L. major *ApoE KO
Final body weight (g)	22.18 ± 0.77	20.67 ± 0.7
Food intake (g/week/mouse)	25.10 ± 0.95	24.72 ± 0.85
Liver lipids		
Total lipids (mg/g)	252.0 ± 12.0	172.0 ± 21.0*
Total cholesterol (mg/g)	14.1 ± 1.3	13.2 ± 0.9
Triacylglycerols (mg/g)	49.7 ± 4.4	30.2 ± 4.5*
Cecum lipids		
Total lipids (mg/g)	51.1 ± 8.9	70.0 ± 11
Total cholesterol (mg/g)	2.8 ± 0.3	2.4 ± 0.3
Triacylglycerols (mg/g)	0.97 ± 0.2	0.86 ± 0.21

Control group, *n* = 8–11, and *L. major *group, *n* = 10–13. Results are expressed as the means ± SE. *P ≤ 0.05.
